# Extreme sediment fluxes in a dryland flash flood

**DOI:** 10.1038/s41598-019-38537-3

**Published:** 2019-02-08

**Authors:** J. M. Hooke

**Affiliations:** Department of Geography and Planning, School of Environmental Sciences, University of Liverpool, Roxby Building, Liverpool, L69 7ZT UK

## Abstract

A flash flood on 28th September, 2012, rose to a peak discharge of 2357 m^3^ s^−1^ from zero within one hour in the ephemeral Nogalte channel in SE Spain. Channel morphology and sediment sizes were measured at existing monitored sites before and after the flood and peak flow hydraulics calculated from surveyed floodmarks and cross-sections. Maximum peak sediment fluxes were calculated as ~600 kg s^−1^ m^−1^, exceeding maximum published, measured dryland channel values by 10 times and common perennial stream fluxes by 100 times. These high fluxes fit the established simple bedload flux - shear stress relations for dryland channels very well, but now extended over a much wider data range. The high sediment fluxes are corroborated by deposits at >1 m height in a channel-side tank, with 90 mm diameter sediment carried in suspension, by transport of large blocks and by massive net aggradation as extensive, structureless channel bars. Very high sediment supply and rapid hydrograph rise and recession produced the conditions for these exceptional sediment dynamics. The results demonstrate the extreme sediment loads that may occur in dryland flash floods and have major implications for catchment and channel management.

## Introduction

## Context

Flash floods in semi-arid areas can be very hazardous and damaging, arising from water flow and inundation but also from the sediment dynamics and impacts of sediment movement. Effects include damage to infrastructure such as roads, bridges, checkdams and embankments, infilling of reservoirs, and occurrence of muddy flows in settlements, all posing major challenges for catchment and channel management. Flow in dryland channels is usually ephemeral and impacts are highly episodic. The large flash floods are also important geomorphologically in producing morphological and sedimentological changes and contributing to longer-term landscape evolution. Here we show the very high sediment fluxes that can occur in such events, the calculated values exceeding previous published recorded values by 10 times.

Process rates of both soil erosion and channel dynamics tend to be high in dryland catchments and sediment yields are amongst the highest globally, particularly in Mediterranean mountainous regions^1,2^. Some sediment flux data on different magnitude events in such channels have been captured from direct measurements at instrumented sites^3–5^, and from incidental observations and post-flood measurements of individual events^6,7^. The common distinctive characteristics of sediment flux in semi-arid, ephemeral channels are: lack of channel bed armour, high sediment supply, and equal mobility of sediment sizes^8^. Sediment fluxes tend to be much higher in ephemeral channels than in humid region, perennial streams, and sediment flux increases very rapidly and simply with shear stress^3–5,9–14^. However, due to the sporadic nature of the flows, their high impacts, and commonly low population density, detailed data in extreme dryland events are still sparse. To increase understanding of the dynamics of flow events and to provide data for validation of modelling of morphological and sedimentological responses to hydrological variations in ephemerally flowing channels^15^, a series of monitored reaches were established on several channels in the semi-arid region of southeast Spain in 1996/7^16,17^.

**Figure 1:**
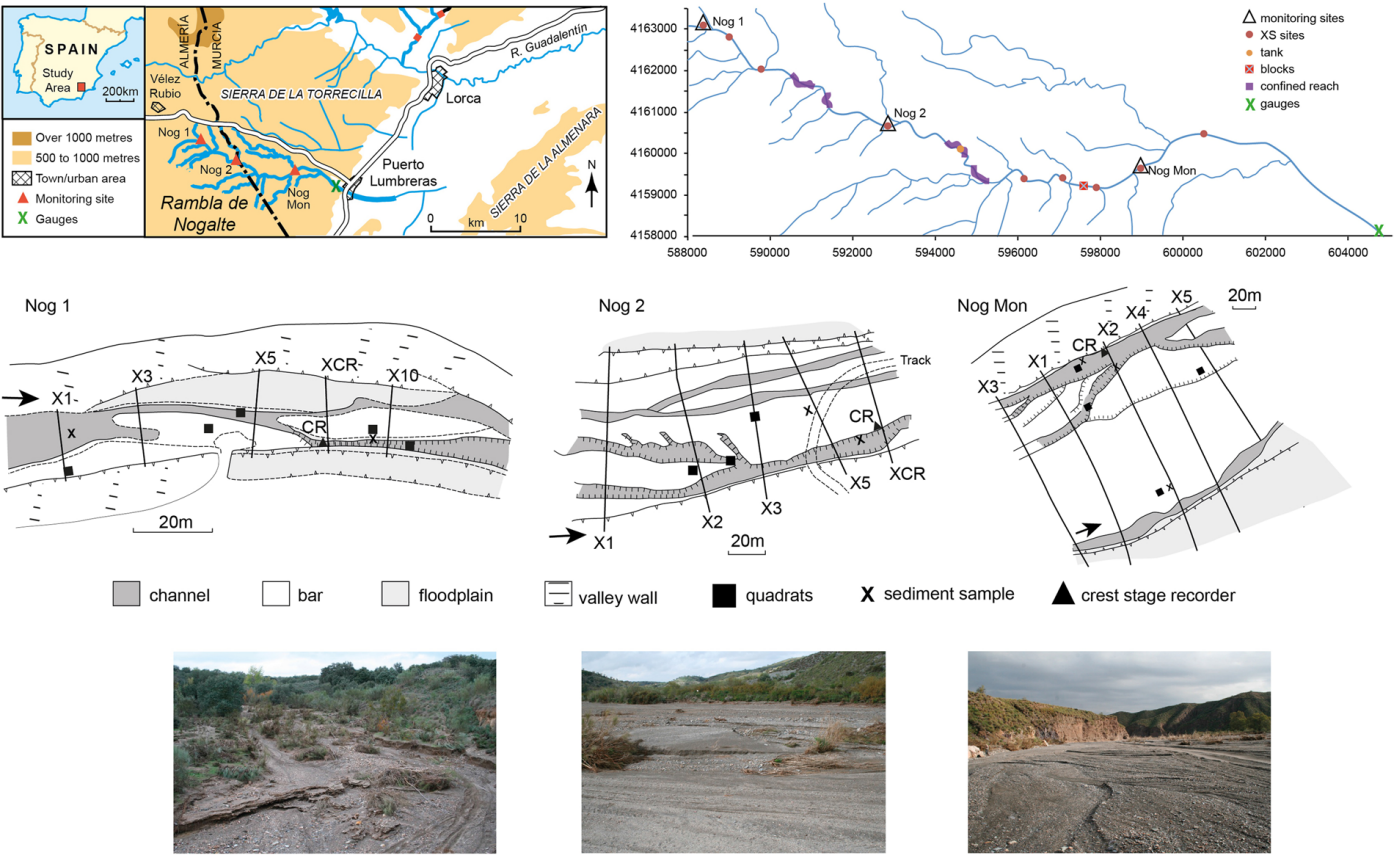
Location and characteristics of field sites: (**a**) location in SE Spain, (**b**) location of measurement sites in Nogalte channel, (**c**) morphological maps of the three study reaches, (**d**) photographs of each of the study reaches.

A major flash flood event, extreme in some aspects, occurred on one of these monitored channels, the Nogalte (Fig. 1), on 28th September, 2012^18^. Prior data on morphology, sedimentology, vegetation and infrastructure state had been measured at the monitored sites^16,17,19^ and repeat surveys were made immediately after the event, with additional data collected on the flood characteristics, processes and channel changes^18^. This paper analyses the evidence and data on sediment dynamics and processes of this high magnitude event and quantifies the sediment flux and flow. Comparisons with world-wide data provide a perspective on characteristics that can occur in such events. Existing formulations are applied to test the extent to which they predict the observed behaviour and sediment transport. This analysis is particularly important because the calculations here of peak flow sediment fluxes have produced record-breaking data.

The Nogalte is tributary to the Guadalentín (Fig. 1), with a catchment area of 137 km^2^ at Puerto Lumbreras where a Confederación Hidrográfica del Segura (CHS) stream gauge is located (604913, 41577776) (Fig. 1). The catchment is set entirely within phyllite schist lithology, with some fluvial gravel terraces. The land use or cover is mainly almond cultivation on the steep valley sides, some grazing of goats, and semi-natural vegetation. *Retama sphaerocarpa* bushes are present in much of the channel. Rainfall averaged 268 mm a^−1^ over the past 10 years. The Nogalte is typical of many gravel ephemeral streams, with a braided pattern along much of its course. Three long-term monitored sites where detailed measurements are made comprise 150–200 m length reaches, located in the upper, middle and lower parts of the main channel (Fig. 1). All have braided morphology but the upper site (Nog1) is much narrower than the middle and lower sites (Nog2 and NogMon) (Fig. 1).

## Event characteristics

**Figure 2:**
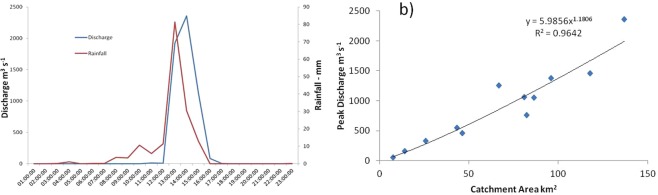
(**a**) Rainfall per hour and discharge measured at CHS gauge at downstream end of Nogalte in event on 28 September, 2012; (**b**) Relationship between calculated peak discharge at surveyed cross-sections and catchment area and gauged peak flow at downstream end of catchment (Puerto Lumbreras).

The rainfall event of 28 September 2012 affected much of SE Spain^20^. Within the Guadalentín basin, the most intense and highest rainfalls were over the Nogalte headwaters and adjacent catchments and resulted in several fatalities as well as severe damage to roads, bridges, bank protection, and irrigational and agricultural structures along the channel edges; regional costs of damage were ~€120 million^21^. Intense rainfall took place after a very hot, dry summer. Total rainfall in the storm was measured as 161 mm over a few hours at Puerto Lumbreras (Fig. 2a) but could have approached 250 mm in the upper Nogalte based on radar images^22,23^ and exceeded 313 mm in Almeria province^24^. Peak rainfall intensities reached 81 mm h^−1^ for an hour at Puerto Lumbreras and the CHS reported maximum daily intensity of 179 l/m^2^, with a peak intensity of 17 l/m^2^ in five minutes^25^. The stream gauge recorded a rise to peak of 2357 m^3^ s^−1^ in one hour and duration to negligible flow of four hours (Fig. 2a)^26^. This exceeds the peak of a devastating flood in 1973 that probably reached 2000 m^3^ s^−1^ ^27^, but implies a flow recurrence interval nearer to 50 years than the 100 year rainfall estimate^23^. The 2012 event peak discharges were calculated from floodmarks surveyed at cross sections within the monitored reaches and down the whole main Nogalte channel soon after the event (Fig. 2b). Calculations using Manning’s roughness coefficient of n = 0.04, adjusted for high Froude Number^28^, provide consistency of values downstream and with the CHS gauged flow at the downstream end^18^ (Fig. 2b). Specific discharges (runoff rates) on the Nogalte attained values comparable with other extreme flash floods in Europe^22,29^, exceeding 100 m^3^ s^−1^ km^−2^ in upper parts of the catchment; the discharge plots above the regional peak discharge against catchment area curve^30^. Flow was continuous throughout the Nogalte system, exhibiting high runoff connectivity.

## Methods

Established cross-sections in each of the three monitored reaches (Fig. 1), topography and multiple floodmarks were resurveyed throughout each reach, using RTK-GPS accurate to ±2 cm. Additional cross-sections were surveyed within the reaches and throughout the catchment main channel (Fig. 2b). All floodmarks were on ground rather than vegetation and convergent values were used to establish the most likely maximum heights. Surveyed floodmarks down the reach on both sides were used to calculate the water surface gradient of the flow. Velocity was calculated using the Manning equation and values of n = 0.04, but adjusted in cases of high velocities and Froude number^28^. The velocity-area method was used to calculate peak discharge and compared with surveys at intervening points down the valley to establish consistency of results and with the gauged outflow. Shear stress, stream power and unit stream power were derived from the measurements (Table 1) and applied in sediment transport and competence equations. All calculations were made using the pre-flood morphology (eight cross-sections in reaches) since large net aggradation took place (with one exception - see Supplementary methods), probably late in the event, in most cross-sections^18^. The MPM (Meyer-Peter-Müller) and Bagnold equations, as the most applicable to these kinds of channels^31^, were used to quantify sediment flux and tested with a range of particle size. Long-established sediment quadrats, 3–5 in each reach, were photographed before and after the flood. Maximum diameter, largest 10 particles (Max 10), and 25 regular grid sampled points (average grid) were parameters measured in each quadrat photograph; representative bulk samples were analysed for each site. DEMs and differences of topography were calculated to produce net change in each reach^18^.

**Table 1:** Calculated peak flow hydraulics at site cross-sections and peak sediment flux calculated using the Bagnold and Meyer-Peter-Muller equations.

Site	Cross section	CS hydraulics								Unit sediment flux qs				
		Width	Depth	Gradient	Velocity	Discharge	Shear stress	Power	Unit power	Bagnold	Bagnold	MPM	MPM	Bagnold-MPM
		m	m		m s^−1^	m^3^ s^−1^	N m^−2^	W m^−3^	W m^−2^	kg s^−1^m^−1^	m^2^ s^−1^	kg s^−1^m^−1^	m^2^ s^−1^	difference
Nog1	**X3**	22.10	0.80	0.024	3.30	58.0	185	13644	617	126.54	0.048	98.3	0.037	12%
	**XCR**	24.31	0.74	0.018	2.41	43.0	108	7699	317	60.21	0.023	52.7	0.020	22%
	**X10**	15.70	0.77	0.029	2.83	44.4	154	12625	621	122.43	0.046	120.5	0.046	2%
Nog2	**X2**	83.20	1.50	0.022	4.50	561.7	287	121107	1456	327.42	0.124	224.4	0.085	31%
	**X3**	76.89	1.80	0.02	4.11	568.2	341	109137	1419	310.19	0.117	242.2	0.091	22%
	**XCR**	79.85	1.65	0.019	4.35	555.4	367	102326	1281	285.02	0.108	203.5	0.077	29%
NogMon	**X1**	129.88	2.17	0.023	5.02	1413.7	478	318655	2453	573.14	0.216	427.9	0.162	25%
	**X2**	135.00	1.76	0.024	4.50	1071.3	413	256180	1898	427.05	0.161	338.9	0.128	21%
**Reach average**														
Nog 1		22.25	0.77	0.0238	2.83	48	147	11254	506	99.8	0.038	87.4	0.033	12%
Nog 2		79.98	1.65	0.0201	4.27	556	327	109657	1371	303.2	0.114	226	0.085	25%
NogMon		132.44	1.97	0.0237	4.76	1238	446	287584	2171	498.1	0.188	385.7	0.146	23%

The floodmark heights and water surface gradients are based on direct and accurate field measurements of position. Uncertainty in likely flood heights measured at each cross-section range from 0.13 m to 1.02 m (Table 2). Water surface slope was calculated from the floodmarks, testing a range of distance around each cross-section. Most probable values were selected from convergent values in lengths and both sides of the channel and consistency of discharge within and between reaches. Possible ranges are indicated (Table 2); uncertainty varies between cross-sections and reaches, ranging up to 33% in likely values. The biggest uncertainty is associated with the choice of Manning’s n value^28^ but 0.04 is consistent with much guidance and mostly produces calculations consistent with the measured flow at the downstream gauge. However, adjustment to 0.05 has been made where Froude number was >1.2, using the method suggested by Lumbroso and Gaume^28^. (Uncertainty associated with choice of Manning n value is indicated in Fig. 8 in Supplementary Methods). Sediment flux was calculated for a range of sediment sizes (d = 5, 10 and 20 mm) but conservative values of hydraulics and of sediment flux (d = 20 mm) are quoted in the results and discussion below. It is suggested from corroborating evidence that these are realistic values for the event.

**Table 2:** Ranges of likely uncertainty for field measured values of flood height and water surface gradient and for derived velocity and discharge values.

Site	Nog1			Nog 2			NogMon	
CS	X3	XCR	X10	X2	X3	XCR	X1	X2
**Flood heights- m elevation**								
Likely value	**840.40**	**839.54**	**839.05**	**743.55**	**743.04**	**741.91**	**601.80**	**600.74**
Max likely	0.10	−0.06	−0.13	0.03	0.00	0.00	0.17	0.00
Min likely	−0.05	−0.07	0.06	−0.69	−1.02	−0.68	−0.69	−0.15
**Gradient**								
Likely value	**0.024**	**0.01825**	**0.029**	**0.022**	**0.0196**	**0.0188**	**0.0230**	**0.0240**
Max likely	0.0006	0.00315	0.001	0.0021	−0.0001	0.0026	0.0081	0.0018
Min likely	−0.0003	0.00005	−0.006	−0.0006	−0.0052	−0.0008	−0.004	−0.0043
**Velocity m s** ^−1^								
Likely value	**3.30**	**2.41**	**2.83**	**4.5**	**4.11**	**4.35**	**5.02**	**4.50**
Max likely	0.04	0.72	0.50	0.00	0.00	0.90	0.31	0.18
Min likely	−0.02	0.00	−0.43	−0.85	−0.28	−0.35	−0.46	−0.10
**Discharge m** ^**3**^ **s** ^−1^								
Likely value	**58.01**	**43.0**	**44.40**	**561.7**	**568.2**	**555.4**	**1413**	**1071**
Max likely	0.7	12.9	1.66	3.8	0.0	37.3	90.0	42.6
Min likely	−0.3	0.0	−11.14	−7.7	−39.0	−23.1	−280.0	−24.1

## Sediment characteristics of deposits

**Figure 3:**
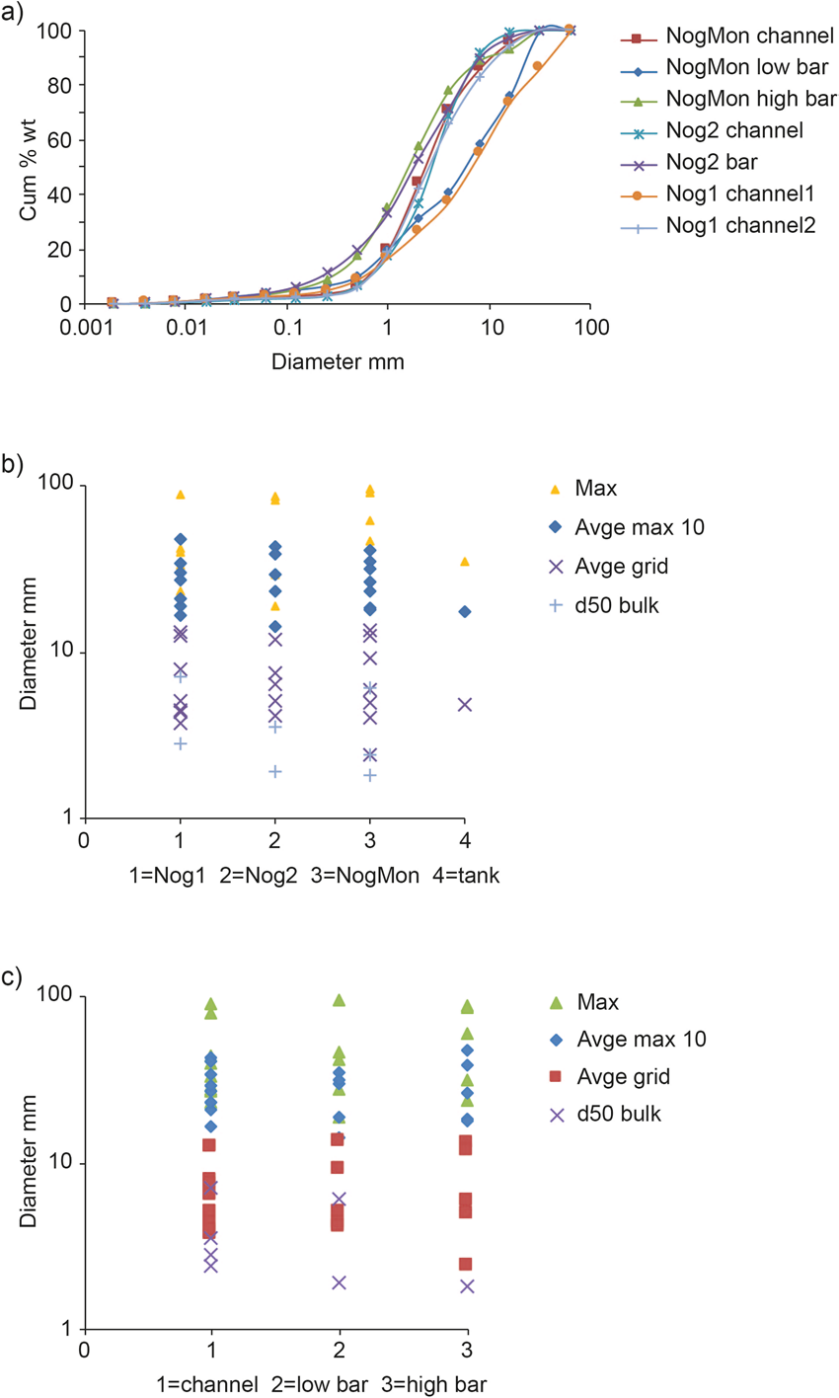
Sediment characteristics of the flood deposits: (**a**) particle size distribution from bulk samples, (**b**) particle sizes from quadrat samples at four sites, (**c**) particle sizes from quadrats at the three monitored sites, classified according to position.

**Figure 4:**
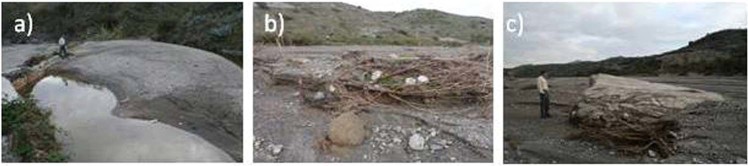
(**a**) Sediment deposited in a channel-side water tank, 1 m above the channel bed; (**b**) coarse particles trapped in vegetation; (**c**) large concrete block transported into the centre of the channel from a wall >250 m away.

The major geomorphological effect was massive deposition as large, flat, unstructured bars in much of the main channel^18^. The channel vegetation was largely destroyed or buried. Net aggradation occurred in all three monitored reaches, to a maximum depth of 0.9 m. Sediment sizes in quadrats, classified by site and by type of deposit (bar or channel) (Fig. 3) indicate maxima of 88 mm, 86 mm, and 96 mm at Nog1, Nog2 and NogMon respectively; average maximum 10 particle size was in the range 14.1–47.5 mm at all three sites, with overall average 28.2 mm. The average grid sample size was 7.3 mm with a range of 2.4–13.4 mm. Bulk samples were only of the finer deposits and give d_50_ ranging from 1.8–7 mm over the three sites. The d_84_ size was 6–9 mm in most samples, and the overall average of d_50_ and d_84_ of the bulk samples are 3.6 mm and 12.7 mm respectively. In the bulk samples 90% or more of the fine fraction is sand so very little cohesive material is present in this system. No clear size distinction is apparent between bar deposits and channels. Ranges and sizes are remarkably consistent between the three reaches, with no downstream fining evident. Sediment was also sampled in a large tank/reservoir at the side of the channel which acted as a sediment trap (Fig. 4a) but had comparable deposits to those in the channel (Fig. 3b). In places in the channel, larger particles of 100–150 mm diameter were deposited within the vegetation (Fig. 4b). Some very large concrete blocks, exceeding 3 m diameter (Fig. 4c) were also were moved a minimum of 250 m into the centre of the channel, at a site upstream of NogMon (Fig. 1).

## Sediment flux

Sediment flux was calculated for each cross-section using MPM and Bagnold sediment transport equations (Table 1), for pre -flood morphology and d_50_ sediment sizes of 5, 10 and 20 mm. Results for d = 20 mm, coarser material than the d_50_ of bulk samples, are presented in order to give conservative, minimal flux estimates. Calculated peak flow fluxes are 60–127 kg s^−1^ m^−1^ (0.023–0.048 m^2^ s^−1^) at Nog1 using Bagnold and 53–121 kg s^−1^ m^−1^ using MPM (0.02–0.046 m^2^ s^−1^), at Nog2 are 285–327 kg s^−1^ m^−1^ (0.108–0.124 m^2^ s^−1^) and 204–242 kg s^−1^ m^−1^ (0.077–0.091 m^2^ s^−1^) for Bagnold and MPM respectively, and are 427–573 kg s^−1^ m^−1^ (0.161–0.216 m^2^ s^−1^) at NogMon using Bagnold and 339–427 kg s^−1^ m^−1^ (0.128–0.162 m^2^ s^−1^) using MPM (Table 1). Maximum differences between Bagnold and MPM estimates are 33% and for d = 0.02 m it is 31%. The maximum flux (at NogMon) is equivalent to 0.57 tonnes s^−1^ m^−1^ or 74 tonnes s^−1^ total flux. These fluxes amount to between 1.1% and 3.5% of the total volume of flow or peak concentrations of 10000–35000 ppm. These maximum load values of the order of 60–600 kg s^−1^ m^−1^ from the upstream to downstream sites, even using coarse material, exceed by an order of magnitude previous measured maxima of 60 kg s^−1^ m^−1^, which is quoted as the published maximum, directly measured bedload flux in ephemeral streams^12^. They exceed values from perennial streams and flumes, commonly used to calibrate sediment transport equations, by two or more orders of magnitude. The results are highly significant in showing what sediment fluxes may occur in a channel, given suitable hydraulic and sediment supply conditions.

**Figure 5:**
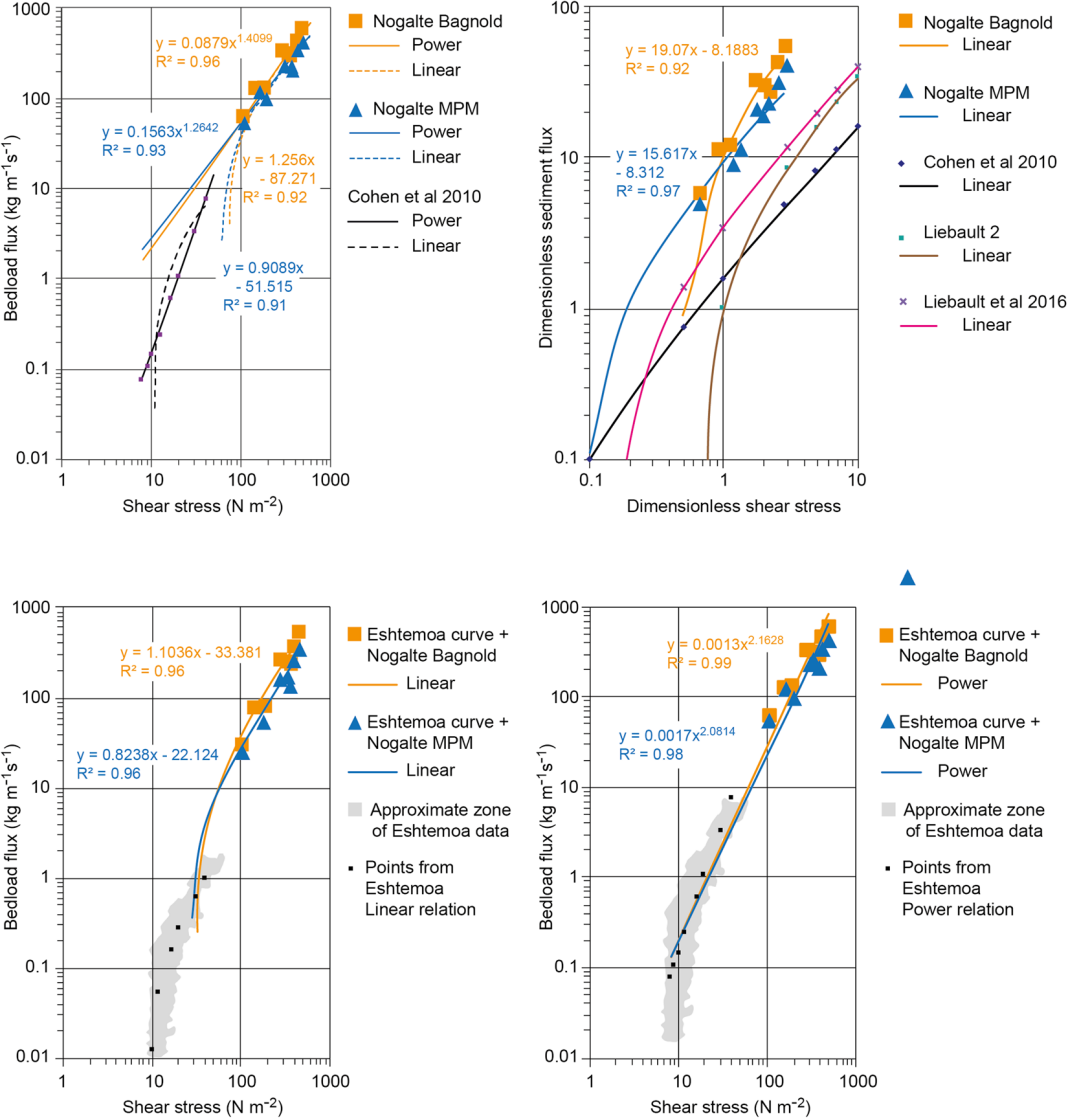
Comparison of calculated Nogalte sediment flux data with published relations of bedload flux to shear stress: (**a**) Nogalte Bagnold and MPM calculated fluxes and shear stress with best-fit linear and power relations and comparison with linear and power relations for Eshtemoa data^13^, (**b**) Dimensionless Nogalte Bagnold and MPM calculated fluxes and dimensionless shear stress with linear relations compared with dimensionless relations of Cohen *et al*.^13^ and Liébault *et al*.^32^, (**c**) Best-fit linear relations of bedload flux to shear stress from combined Nogalte data and Eshtemoa curve^13^, (**d**) Best-fit power relations of bedload flux to shear stress from combined Nogalte data and Eshtemoa curve^13^.

If the data calculated here are added to the relations established for instrumented sites in Israel at which the published maxima were measured^11–13^, then the peak sediment fluxes fit those relations to shear stress very well (r^2^ > 0.96), confirming the simple relation but extending its application by an order of magnitude (Fig. 5) (though the power relations fit slightly better than the linear, in contrast to Cohen *et al*.^13^). The dimensionless rates are higher than the record relations published hitherto^13,32^. The Nogalte Bagnold data combined with the Eshtemoa power relation for that data range produce a correlation coefficient of r^2^ = 0.99. The calculated Nogalte MPM values incorporate shear stress so a strong relation is expected but the Bagnold calculations use unit stream power and velocity. The Bagnold relations produce higher correlations than those of MPM, both for the Nogalte data and the combined Eshtemoa relation.

## Competence, mode of transport and sediment budget

**Figure 6:**
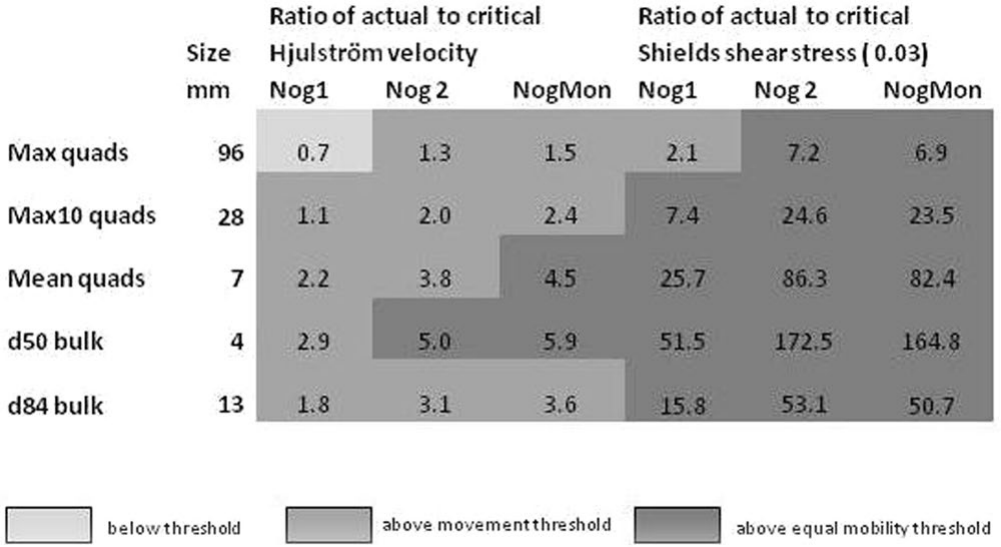
Ratios of actual to critical values at each site for movement of average sizes of sediment measured in quadrats and bulk samples.

**Figure 7:**
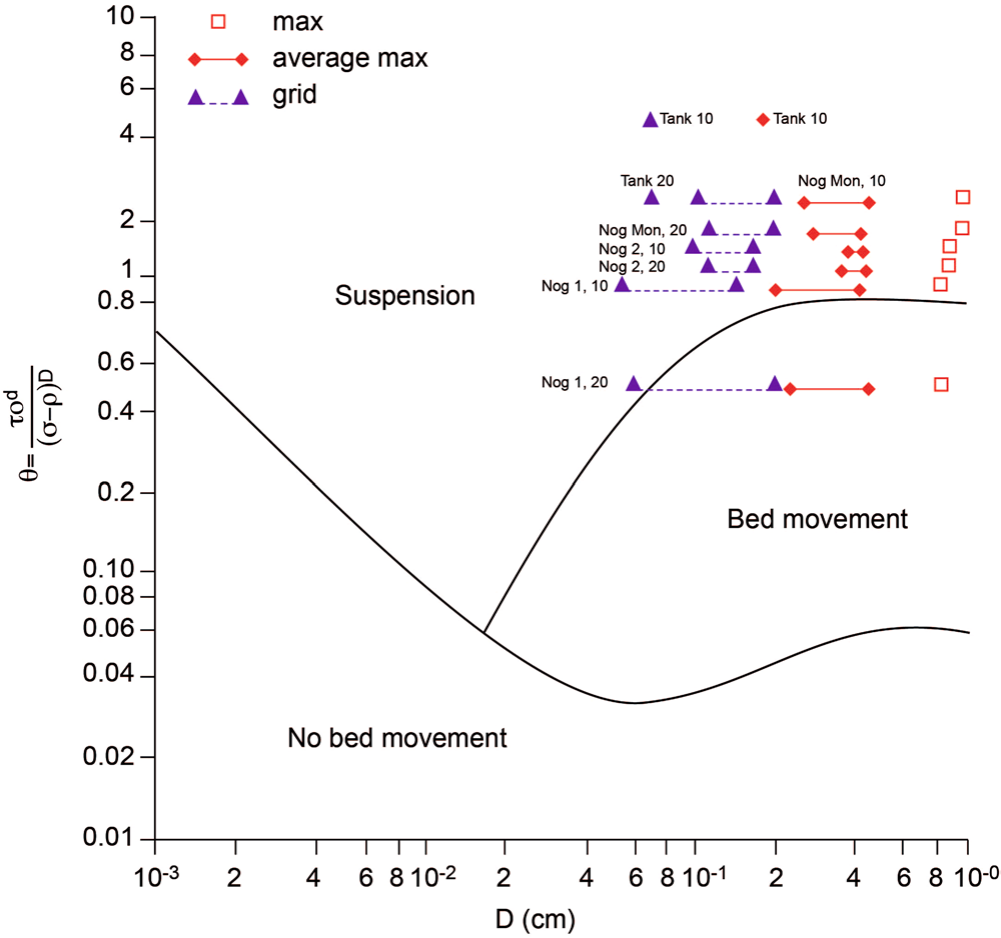
Calculated Shields entrainment function using D = 10 and D = 20 for each site for measured sediment parameters: maximum size in a quadrat (max), average of 10 largest particles in a quadrat (Max 10), average of regular grid sample of size in quadrat (grid). The ranges indicate the values measured at the multiple quadrat sample points at each site. Source of base graph: Embleton and Thornes^35^.

The sizes of sediment deposited can be used to assess the competence and transport mechanisms in the event and applied to competence formulations. Using both the Hjulström velocity curve^33^ and Shields shear stress^34^ to calculate competence of the flows, the values at peak flow in all sites exceed their predicted thresholds for movement of all quadrat and bulk sample particles sizes (Fig. 6). The ratios of actual to critical values of velocity range up to 3.6 for the d_84_ of samples at sites. The ratio of actual shear stress to Shields critical values, using 0.03 loose bed, ranged between 52 for 4 mm particles to 1.4 for 150 mm particles at Nog1, and at Nog2, and ranged between 170 for 4 mm particles and 1.7 for 400 mm particle at NogMon. Values exceed the 4.5x critical Shields shear stress, identified for equal mobility^35^, for all quadrat sample sizes at all sites. Calculations for surveyed cross-sections elsewhere in the channel course (Fig. 1) indicate all exceeded the sediment movement thresholds so transport of all sizes took place throughout the channel system. Evidence of the mode and mechanism of transport is provided by the Shields values for d = 10, 20 and 32 mm (as conservative values) for the various sediment size parameters in the quadrats at each site (Fig. 7)^36^. Assuming d_50_ bed material even up to 20 mm, then at Nog2 and NogMon all sizes of material would be in suspension, and at Nog1 all coarse material would be moving as bedload with fine material in suspension. For a bed material of 32 mm all material would be moving as bedload; however, 32 mm is in the range of the maximum 10 particles sampled by the quadrats so is at the extreme of the sediment sizes. The pre-flood bed was not armoured by these very large particles and the post-flood bed was very loose and unstructured.

Additional evidence of the size of material transported during the event is from deposits in a large, 45 × 15 m area, water storage tank/reservoir located at the channel side in the confined, middle part of the course, between sites Nog2 and NogMon (Fig. 1), and beyond a wall 1–1.3 m high above the channel bed, which acted as a sediment trap in the event (empty beforehand) (Fig. 4a). The sizes deposited are exactly comparable with the other channel deposits (Fig. 3), with a maximum sampled quadrat size of 35 mm, but particles of 82 mm were present on the tank deposit surface, 1.3 m above the channel bed. The values of Shields parameter for the tank indicate that the material was easily carried in suspension (Fig. 6). Elsewhere throughout the channel, many particles of 100–150 mm diameter were deposited within the vegetation, indicating movement at height (Fig. 4b). In addition, some very large blocks were moved in a few locations, including two concrete blocks found in the centre of the channel upstream of NogMon (Fig. 1), which were 7.2 m long, with b axis of 3–4 m (Fig. 4c). These concrete blocks must have come from channel walls ~300 m away. Many competence formulae fail to predict movement of blocks of this size under these hydraulic conditions. Overall, the competence measurements corroborate the evidence of high sediment flux and sediment mobility.

Deposition was extensive over most of the active channel area, which occupied much of the valley floor in all three sites. Using difference of pre- and post-flood DEMs, the net change is the equivalent of 108 m^3^ of sediment added in Nog1 reach, 1548 m^3^ in Nog2, and 5648 m^3^ in NogMon^18^. Sediment budget calculations indicate that this material must have been derived directly from slope sources as a net addition during the event. Comparison of six bulk samples of the bare soil in the almond groves on the slopes (4 samples) and under semi-natural vegetation (2 samples) with seven bulk samples of channel sediment shows little coarsening of the channel deposits: 58.9% gravel compared with 62% in the soil, 38.2% sand compared with 31.5% in soil, and 3% silt-clay compared with 6.5% in the soil, confirming the slope erosion as the probable direct source.

## Wider comparison and implications

Little change in size of surface material occurred as a result of the flood in spite of high mobility and completely new surfaces at a different elevation created over much of the active channel area. The sediment deposits are extremely loose and lacking resistant or armoured surfaces and the sediment lacks fine and cohesive material. The similarity of deposits throughout the system implies a similarity of both supply and of hydraulics, a lack of sorting and rapid deposition; the channel material is similar in particle size to the sampled slope materials. Coarse material (>100 mm diameter) is sparse. The schistose bedrock breaks up very easily to relatively homogeneous gravel-sized particles and is easily transported.

The sediment budget calculations indicate a large addition of material to the main channel system to account for the amount of net deposition, which was almost continuous along the channel. If the net deposition gain rate at Nog2 of 700 m^3^ per 100 m length is applied to the 5 km length of channel with similar, wide aggrading morphology between Nog2 and NogMon, then the net gain of material is of the order of 40,000 m^3^ of material (65,500 tonnes). If that is coming from the catchment area between Nog2 and NogMon (60 km^2^) the yield becomes 1091 tonnes km^−2^ or 661 m^3^ km^−2^. This is equivalent to 0.66 mm erosion over the whole surface in one event of a few hours duration and equivalent to some of the highest published annual sediment yields globally^32^.

Overall, the evidence is of an event with an extremely high rate of hydrograph rise, in which all the deep, loose gravel channel material across nearly the whole valley floor was mobilised immediately. Supply of sediment from the slopes was very high due to fissile, schistose material, the high unit runoff and the dominantly bare, steep slopes under almond groves throughout the valley. This produced very large sediment loading which prevented erosion taking place early in the event; the rapid recession, still with high sediment loads, meant little net erosion occurred in late stages. The massive, unsorted load was rapidly deposited on the sharp recession, leaving large, flat planar bars. The liability to high sediment fluxes is corroborated by the catchment authority’s (CHS) assessment of the catchment risks and dangers associated with floods, who have rated the level of sediment transport risk at the very highest level of 5/5^37^. The calculations here confirm that assessment.

This event was a major hazard to human life and produced significant infrastructure damage. The natural channels are well adapted to carrying these fluxes, and most problems of structural damage occurred where channels were constrained by walls and embankments. Management strategies must allow room for the flow and channel mobility and for these possible sediment fluxes. The large sediment flux also has major implications for filling of checkdams; the flux was in spite of many small checkdams being present in the catchment, though some were full. Since the flood event many more, large checkdams have been constructed as the major strategy for reducing flooding downstream but their capacity and longevity could be reduced quickly if similar events occur. Future climate scenarios could exacerbate these problems. The data presented here indicate the magnitude of sediment fluxes that are possible in ephemeral flash floods, given high sediment supply and intense hydraulic conditions.

## Methods

### Evidence and field measurements

Routine measurements at the monitored sites include: peak flow by crest stage recorder; topography by detailed RTK-GPS surveys of cross-sections, thalweg, all channel edges, and points across the channel and floodplain; sediment size and surface characteristics by photography of established 0.5 m quadrats; and vegetation cover, state, species and height by survey of established 3 m quadrats^16^. Changes are measured by comparison of repeat surveys after flows^16,17^ from which DEMs have been constructed and the DoDs for each reach calculated for changes in major events^18,38^.

**Figure 8:**
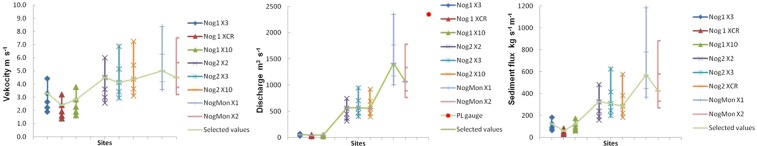
Values of velocity, discharge and Bagnold sediment flux for cross-sections, indicating uncertainty associated with values of Manning’s n from 0.03 (maximum values plotted) to 0.07 (minimum values plotted).

Peak discharge and hydraulics (velocity, shear stress, stream power and unit stream power) of the event have been calculated from surveyed floodmarks in each of the three monitored reaches, combined with the surveyed cross-sections before and after the event, and surveyed points throughout the reach^18^ (Fig. 2b). RTK-GPS points are measured to an accuracy of ±2 cm. Hourly discharge data during the event (Fig. 2a) were also available from the CHS website^26^ for the gauge at Puerto Lumbreras town, further downstream (Fig. 1). (No higher temporal resolution data are available but the rapidity of the recession means that the peak is unlikely to have been much higher than this validated value). Hydraulics have been calculated for 3–5 post-flood cross-sections in each reach (Fig. 1) using the velocity-area method and testing with a range of Manning’s n values and uncertainty in gradient and floodmark levels. Convergent values have been selected as most probable. Hydraulics have been calculated for both pre- and post-flood morphology for those cross-sections surveyed immediately before the flood and pre-flood hydraulics are used here (Table 1) because of the high sedimentation at most locations, which probably occurred post-peak (with the exception of Nog1 X10 where the prior survey was some time beforehand and the section was erosional so post-flood morphology was used). The floodmark heights and water surface gradients are based on direct and accurate field measurements of position. Uncertainty in likely flood heights measured at each cross-section range from 0.13 cm to 1.02 m (Table 2). Water surface slope was calculated from the floodmarks throughout the reach to gain water surface profiles, testing a range of distance around each cross-section. Most probable values were selected from convergent values in lengths and both sides of the channel and consistency of discharge within and between reaches. Possible ranges are indicated; uncertainty varies between cross-sections and reaches, ranging up to 33% in likely values. The biggest uncertainty is associated with the choice of Manning’s n value but 0.04 is considered suitable as an initial test and mostly produces calculations consistent with the measured flow at the downstream gauge for post-flood morphology^18^ and for intervening cross-sections measured throughout the system^18^ (Fig. 2b), but for Froude Number values of >1.2 adjustments were made (three cases) in accordance with analysis in the HYDRATE project^28^. The uncertainty arising from selection of Manning n value is illustrated in Fig. 8 for velocity, discharge and Bagnold sediment flux values using a range of n from 0.03 to 0.07. Overall, all the evidence combined and the calculations of uncertainty indicate that values in the event are unlikely to be lower than those used and thus the calculated sediment flux is probably a conservative estimate.

The long-established, sediment quadrats in fixed horizontal position, 3–5 in each reach, were photographed before and after the flood, with no intervening flow taking place between pre- and post-flood measurements. From the images, several size parameters have been measured in each quadrat - maximum, largest 10 particles (Max 10), and mean and standard deviation of 25 regular grid sampled points; changes in each from pre- to post-flood were also calculated. In addition, some representative bulk samples were taken and analysed in the laboratory using sieving for particles >2 mm and Coulter Counter for <2 mm size. Bulk samples were also taken of soil material on the slopes to assess characteristics of potential supply. At the most downstream site, NogMon, pits were dug in the channel bed after the event to examine evidence of stratigraphy, layering and grading, and of the depth of the active layer in the event.

In addition, cross-sections at other locations through the whole channel system were surveyed after the flood to calculate peak discharge and hydraulics^18^. Observations and mapping of channel features, evidence of erosion and deposition, sizes of sediment, and nature of tributary supply were conducted along the whole 25 km length of channel. This included hydraulic and sediment measurements at a site where a tank or small reservoir had acted as a sediment trap in the channel and a location where some very large blocks had been deposited (Fig. 1). These data provide corroboration for some inferences about dynamics of the event.

Volumes of sediment eroded and deposited and net sediment budget within the three monitored reaches have been calculated from the pre- and post-flood DEMs constructed from the detailed topographic surveys. Calculations of the difference of DEMs (DODs), using ArcGIS (10.4.1) with the TIN algorithm and the ‘Geomorphological Change Detection’ plug-in (GCD 6) procedures attached to ArcGIS, were used to calculate net sediment volume changes and uncertainties. The volumes derived from the DoDs have been used to calculate the net sediment flux using the morphological method^39,40^.

### Derived calculations and modelling of sediment dynamics

The following aspects of the sediment dynamics have been analysed and modelled: sediment flux, flow competence, mode of transport, sediment yield and sediment budget. For sediment flux or sediment transport load, a wide number of equations for calculation of sediment flux or amount transported have been developed, very many from flume experiments and using homogenous sediment, and most of the other field-derived relations are from very controlled and perennial flow channels. Most have encompassed relatively small ranges of shear stress values. It is notorious that use of various sediment questions produces very wide variations of estimates of load, especially bedload, often one or more orders of magnitude^40^. Some equations are for bedload only, others for suspended sediment and some for total (combined load); some use mixed sediment sizes^41^. Reid and associates^8^ have investigated the applicability of various equations in relation to field measurement data from the Israeli instrumented ephemeral channels and have found that the Meyer-Peter and Müller^42^ equation fits the Nahel Yatir and Eshtemoa bedload data collected over several years reasonably well. Gomez and Church^43^ state that stream power equations provide the most straightforward scale correlation of flow and sediment transport and should be used when information on channel hydraulics is limited and Graf (1988, p149)^44^ advocated the use of the Bagnold^45^ total load equation by geomorphologists. The Bagnold equations are based on stream power and were used, for example, by Graf^46^ in his model of sediment movement in the ephemeral streams near Los Alamos, USA. It also worked well in a model developed to simulate morphological, sediment and vegetation processes for these present studied channels in SE Spain^16^. In the present study, various sediment transport equations were tested including MPM loose^42^, MPM Parker^47^, MPM Wilson^48^ variation, and Bagnold total load^45^, but results are focused on the Bagnold (equation 1) and MPM equations (equation 2)^48^, which gave reasonably convergent results (Table 1) and because they have been found to be highly applicable to measured loads in ephemeral stream floods^31^. Equations:

Bagnold^45^

1 $$i={\rm{\omega }}(\frac{{e}_{b}}{\tan \,{\rm{\alpha }}}+0.01\frac{u}{{v}_{{\rm{ss}}}})$$

where *i* is total load transport (kg s^−1^), ω is unit stream power (N m^−1^ s^−1^), *e*_b_ is a bedload efficiency factor, tan α is coefficient of friction, *u* is flow velocity (m s^−1^), *v*_ss_ is settling velocity of particles for a given size (m s^−1^). Efficiency and tan α values are as tested in Hooke *et al*.^15^.

MPM^49^

2 $${{\rm{q}}}_{{\rm{b}}}={{\rm{\O }}(({\rm{\rho }}}_{{\rm{s}}}\,-\,{\rm{\rho }})\,{\rm{g}}\,{{\rm{d}}}^{{\rm{3}}}{)}^{{\rm{0.5}}}$$

where

$${\rm{\O }}={((4{\rm{\tau }}/{\rm{\rho }}({{\rm{\rho }}}_{{\rm{s}}}-{\rm{\rho }}){\rm{gd}})-0.188)}^{1.5}$$

ρ_s_ is sediment density (kg m^−3^), ρ is fluid density (kg m^−3^), g is gravitational acceleration (m s^−2^), d is grain diameter (m), and τ is shear stress (N m^−2^)^49^.

All calculations have used 0.7 porosity or 1.65 density to convert weight to volume. An efficiency value of 0.15 and fall velocity values of 0.3–0.9 were used for the Bagnold equation (1). All calculations have been made for d_50_ = 10 mm and 20 mm to give conservative quantities and for d = 5 mm which is near the d_50_ of bulk samples, which are biased towards fine material. Results are presented using the Bagnold total load equation (1) and the MPM equation (2). Uncertainty in the Bagnold estimates are affected by velocity (and thus discharge and unit stream power) and effects of Manning n values are indicated in Fig. 8.

The data derived from the Bagnold and MPM calculations of peak sediment flux in each cross–section were compared with the published relations between bedload flux and shear stress^11–13^, which show a simple relation, though for smaller magnitude values. The Nogalte values have been plotted with best-fit linear and power relations together with the separate curves established by Cohen *et al*.^12^ for the whole Eshtemoa data set (Fig. 5a). The Nogalte values and best-fit relations have also been plotted for dimensionless values together with published relationships^12,32^ (Fig. 5b). In addition, the best-fit linear and power relationships have been calculated for the combined Nogalte data and Cohen *et al*. curves (using calculated points, not the original whole Eshtemoa dataset) (Fig. 5c,d). All calculations were made using excel.

The sediment budget has been calculated from the sediment continuity equation of input-output-change model using calculations of flux calculated as above and evidence of amounts of erosion, deposition and net change in a reach from the DoDs and cross-sections. Sediment yield produced by this event has also been calculated from the data of flux and net storage. No direct sediment load measurements (suspended or bedload) are available for the gauging station or elsewhere. In such an event, standard measurement techniques for suspended load are unlikely to be meaningful anyway since the evidence is of very coarse particles being carried in suspension. It is suggested that the detailed field measurements made here, combined with the methods of calculation of fluxes and the corroborating evidence give estimates that are realistic for the event.

Many equations and relations are available for calculating competence and Buffington and Montgomery^50^ reviewed analyses to that date. Hooke *et al*.^15^, in developing a simulation model of morphological, sedimentological and vegetation changes in these channels, tested various formulations in terms of critical velocity, critical shear stress, critical discharge, critical depth and critical power. Following this review, they used the values derived from the Hjulström graph for entrainment and the lower velocities at which sedimentation takes place for any size, though the Baker and Ritter^51^ equation was also considered. More recently, Billi^52,53^ has tested several critical shear stress equations on ephemeral channels in Ethiopia, including for predictions of the entrainment of large boulders. Thompson and Croke^54^ also reviewed and tested eight competence equations. In assessing competence of later, very large floods in perennial rivers in Queensland^55^, they tested Costa’s^56^ critical unit stream power regression model, D_reg_, and the empirical lower envelope curve for critical stream power, D_lec_, as well as the Shields entrainment function, and found the D_reg_ equation did not predict entrainment of the 2 m boulders transported but the D_lec_ equation did.

In the current analysis a range of equations and relations for competence were tested^33,34,52,57–62^. A calculator created by Mecklenburg and Ward^49^ was also used to check the Shields entrainment function. The competence values were calculated for the actual hydraulics at each cross-section to predict the size that could be moved. The hydraulics necessary for mobilisation of the mean and maximum sizes and for d = 5 mm, d = 10 mm and d = 20 mm have also been calculated. In addition, competence and required hydraulics (velocity and shear stress) to transport some very large blocks were calculated. Ratios of actual hydraulic values in each section to critical values for the range of sizes found were calculated (Fig. 6)^35,59^.

## Data Availability

The datasets generated during and/or analysed during the current study are available from the corresponding author on reasonable request.
